# Abdominal Massage Reduces Visceral Hypersensitivity via Regulating GDNF and PI3K/AKT Signal Pathway in a Rat Model of Irritable Bowel Syndrome

**DOI:** 10.1155/2020/3912931

**Published:** 2020-06-05

**Authors:** Bo Li, Xiong-Fei Luo, Si-Wen Liu, Na Zhao, Hua-Nan Li, Wei Zhang, Ying-Ying Chen, An Bao, Jin-Gui Wang, Qiang-Song Wang

**Affiliations:** ^1^First Teaching Hospital of Tianjin University of Traditional Chinese Medicine, Tianjin 300193, China; ^2^Institute of Biomedical Engineering, Chinese Academy of Medical Science & Peking Union Medical College, Tianjin 300192, China

## Abstract

Changes in gut motility and visceral hypersensitivity are two major features of irritable bowel syndrome (IBS). Current drug treatments are often poorly efficacious, with many side effects for patients with IBS. Complementary therapies, such as acupuncture or abdominal massage, have received more attention in recent years. In this study, a rat model of IBS with diarrhea (IBS-D) was established by instillation of acetic acid from the colon. The effects of abdominal massage on changes in gut motility, visceral hypersensitivity, and the possible mechanism were investigated. Continuous abdominal massage could decrease the stool consistency score and increase the efflux time of glass beads compared with model groups, while also decreasing mast cell counts in IBS-D rats. The mRNA and protein expressions of neuronal nitric oxide synthase (nNOS), choline acetyl transferase (CHAT), and protein gene product 9.5 (PGP9.5) were significantly upregulated by continuous abdominal massage compared with model groups. Continuous abdominal massage also improved the ultrastructure of enteric glial cells (EGCs) by decreasing the number of mitochondria and increasing the level of the heterochromatin. Meanwhile, continuous abdominal massage could upregulate the expression of glial cell line-derived neurotrophic factor (GDNF) and P-Akt/Akt. Furthermore, it could reduce visceral hypersensitivity and improve the IBS-D symptoms by regulating the phosphoinositide 3-kinase (PI3K)-Akt pathway, which would provide a novel method for the treatment of IBS-D in the clinical setting.

## 1. Introduction

Irritable bowel syndrome (IBS) is a functional disorder characterized by chronic recurring abdominal pain or discomfort associated with disordered bowel habits [1]. Approximately 4%–20% of adults and adolescents throughout the world exhibit symptoms consistent with IBS, and there is a female predominance in the clinic [2, 3]. IBS with diarrhea (IBS-D) was the most common IBS in China [4], impairing the quality of life of sufferers, which conferred a heavy economic and social burden on the society. The pathophysiology of the functional bowel disorder is poorly understood, and a lack of biomarkers has resulted in difficulty in diagnosis and targeted therapy [5]. Therefore, effective drug treatment was also hampered by the absence of reliable biologic markers and defined end points for IBS [6]. These days, complementary therapies, including acupuncture or abdominal massage, are receiving more attention for treating IBS [7, 8].

It has been reported that the enteric nervous system (ENS) and its neurotransmitters play an important role in altering gastrointestinal (GI) functions [9]. ENS neurons include inhibitory neurons, such as neuronal nitric oxide synthase (nNOS) and excitatory neurons, such as CHAT. Enteric glial cells (EGCs) are the main components of ENS and are essential for the control of gastrointestinal functions. Glial cell line-derived neurotrophic factor (GDNF) is an important neurotrophic factor for ENS, which is related to regulate the proliferation, maturation, and survival of the enteric neurons [10]. GDNF could activate the RET tyrosine kinase receptor, resulting in the stimulation of the phosphoinositide 3-kinase (PI3K) pathway, which is the main signaling pathway for GDNF [11]. Activating the PI3K pathway could result in the phosphorylation of Akt (p-Akt), which is the major downstream target of the PI3K pathway.

Our previous studies have demonstrated that abdominal massage has beneficial effects in the treatment of chronic fatigue syndrome [12], during which the effect of abdominal massage on IBS was first reported, and the underlying biological mechanism was still unknown. In this study, a rat model of IBS-D was established, and continuous abdominal massage was performed with treatment of IBS-D. The protein expressions of CHAT, nNOS, and PGP 9.5 were investigated by immunofluorescence and western blot, respectively, and the mRNA expression levels of CHAT, nNOS, and PGP 9.5 were analyzed using real-time RT-PCR. Furthermore, the ultrastructure of enteric glial cells (EGCs) was observed by transmission electron microscopy (TEM), and the PI3K-Akt signaling pathway was investigated by western blot. The present study provides a novel perception of the mechanism of abdominal massage for the treatment of IBS, especially for IBS with diarrhea.

## 2. Materials and Methods

### 2.1. Reagents and Animals

The mammalian cell lysis kit (Pierce BCA protein assay kit) was purchased from Thermo Fisher Scientific (Waltham, MA). The UNIQ-10 column Trizol total RNA extraction kit, anti-GAPDH, and peroxidase-conjugated secondary antibody were purchased from Sangon Biotech, Co., Ltd. (Shanghai, China). Anti-PGP 9.5, anti-nNOS, and anti-CHAT were purchased from Thermo Fisher Scientific (Waltham, MA, USA). The First Strand cDNA Synthesis Kit and FastStart Universal SYBR Green Master (ROX) kit were purchased from Roche (Mannheim, Germany).

Male Sprague Dawley (SD) rats (180–220 g) were obtained from Beijing Vital River Laboratory Animal Technology Co., Ltd. (Beijing, China). The animals were permitted free access to standard food and water under standard temperature (24°C), relative humidity (50%), and light (light and dark cycles). Animal experiments were performed according to the National Institutes of Health Guide for Care and Use of Laboratory Animals, and the protocols were approved by the Ethics Committee of Tianjin University of Traditional Chinese Medicine.

A rat model of the postinfectious irritable bowel syndrome induced by acetic acid was performed by reference of Liu et al. [13] with slight modifications. Abdominal massage was performed according to our previous method [14]. In brief, the power of abdominal massage was first corrected by the massage manipulation simulator (China patent: ZL200710187403.1). The abdominal massage was performed on acupoints of Guan Yuan and Zhongwan for about 6 minutes with one treatment per day and continued for a total of 14 days.

### 2.2. Stool Consistency Score, Efflux Time of Glass Beads, and Abdominal Retraction Reflex (AWR) Score

The stool consistency score was determined using the Bristol Stool Form Scale (Table S1) [1]. The rats were then administered glass beads (3 mm in diameter) from the anus to the rectum (about 3 cm) under anesthesia. The rats were kept with free access to standard food and water, and the efflux time of glass beads was calculated. Colorectal distension (CRD) was performed as previously described, and the AWR scores of rats were calculated (Table 1) [15]. In brief, a latex double-lumen catheter attached to a balloon dilator was inserted into the colon with the distal tip locating at the site 6 cm away from the anus. The rats were placed into small lucite cages and kept to wake them up for 30 min. CRD was maintained with water injection and repeated 3 times to achieve an accurate measurement. Colorectal distension (CRD) was calculated as the amount of injected water when the AWR score was 3.

### 2.3. Histological Assessment and Mast Cell Counts

After the treatment, rats were aestheticized by injecting chloral hydrate (i.p., 400 mg/kg), and the colons were resected quickly and washed with ice PBS. All steps were performed under icy conditions. Distal colons were isolated and fixed in 10% buffered formalin solution followed by dehydration in a graded ethanol series followed by xylene. Samples were embedded in paraffin, cut into thin sections of 5 *μ*m thickness, and stained with hematoxylin and eosin (H&E) and toluidine blue. The stained sections were observed using light microscopy.

### 2.4. Immunofluorescence of CHAT, nNOS, and PGP 9.5

The fixed slides were deparaffinized and rehydrated. 4′6-diamidino-2-phenylindole (DAPI) was performed to observe the cell nucleus (blue) and immunofluorescence staining using CHAT, nNOS, and PGP 9.5 antibodies and immunofluorescence detection kit from Sangon Biotech, Co., Ltd., (Shanghai, China). Fluorescence of the tetramethylrhodamine isothiocyanate- (TRITC-) marked secondary antibody (red) in slice tissues was observed under a fluorescence microscope with a wavelength of 490 nm.

### 2.5. Effects of Abdominal Massage on the mRNA and Protein Expression of CHAT, nNOS, and PGP 9.5

Total RNA was extracted from colon tissues using the Sangon UNIQ-10 column Trizol total extraction kit according to the manufacturer's instructions. cDNA was obtained using an ImProm-II reverse transcription system under the following conditions: 37°C for 15 min, 85°C for 5 s, and 4°C. The real-time RT-PCR oligonucleotide primers are shown in Table S2. Amplification was performed with an ABI Prism 7500 real-time PCR system as follows: 95°C for 30 s, 40 cycles of 95°C for 5 s, and 60°C for 31 s. A melt curve analysis was performed at the end of each experiment. The mRNA expression of samples was analyzed using the 2^−ΔΔCT^ method with *β*-actin as an internal control [16, 17].

The protein expression levels of CHAT, nNOS, and PGP 9.5 were analyzed by western blot. The total protein was extracted using a mammalian protein extraction kit (Sangon, China), and the protein concentration was detected by the BCA method (Pierce, USA). Protein (30 mg) was electroblotted onto a PVDF membrane following separation on an 8% SDS-polyacrylamide gel. The PVDF membrane was incubated with blocking solution (5% skim milk) for 2 h and overnight with a 1 : 500 dilution of anti-CHAT, anti-nNOS, anti-PGP 9.5, and anti-GAPDH antibodies at 4°C. Blots were washed and incubated with a 1 : 2,000 dilution of horseradish peroxidase-conjugated secondary antibody. Blots were washed five times with TTBS again and then developed using a horseradish peroxidase substrate and captured on a ChemiScope 6200 chemiluminescence imaging system (Clinx Science Instruments Co., Ltd.).

### 2.6. Effects of Abdominal Massage on the Ultrastructure of Enteric Glial Cells (EGCs)

The fixed colon tissues were dehydrated in a graded ethanol series followed by acetone. Samples were embedded in epoxy resin and cut into thin sections of 1-2 *μ*m thickness. The sample was stained with toluidine blue to search for the specific area, and then the sample was cut into 50–70 nm thickness on a copper grid. The sample was stained with uranyl acetate to obtain the ultrastructure of enteric glial cells by transmission electron microscopy using the Hitachi TEM system from Hitachi High-Tech CO. (Tokyo, Japan).

### 2.7. Effects of Abdominal Massage on the PI3K-Akt Signal Pathway

The effects of abdominal massage on the PI3K-Akt signal pathway were investigated by western blot. All proteins were extracted using a mammalian protein extraction kit (Sangon, China), and the protein concentration was detected by the BCA method (Pierce, USA). The 30 mg protein was electroblotted onto a polyvinylidene fluoride (PVDF) membrane following separation on an 8% SDS-polyacrylamide gel. The PVDF membrane was incubated with blocking solution (5% skim milk) for 2 h and overnight with a 1 : 1,000 dilution of anti-GDNF, anti-P-Akt, and anti-GAPDH antibodies at 4°C. Blots were washed and incubated with a 1 : 2,000 dilution of the horseradish peroxidase-conjugated secondary antibody. Blots were washed five times with TTBS again and then developed using a horseradish peroxidase substrate and captured on a ChemiScope 6200 chemiluminescence imaging system (Clinx Science Instruments Co., Ltd.).

### 2.8. Statistical Analysis

All statistical analyses were performed using Origin Pro 8.5 software from OriginLab (Northampton, MA), and the results are presented as the mean ± SD. Statistically significant differences between groups were determined by one-way analysis of variance (ANOVA). A value of *P* < 0.05 was considered statistically significant, and *P* < 0.01 was considered to be highly significant.

## 3. Results

### 3.1. Effects of Abdominal Massage on Intestinal Transit and Visceral Sensitivity of Rats

**Figure 1:**
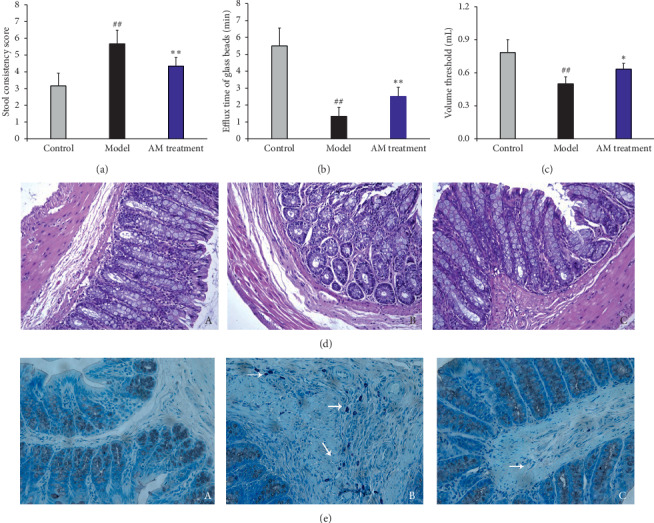
Effects of abdominal massage on intestinal transit and visceral sensitivity (a–c), colonic histological assessment, and mast cell counts of IBS-D rats. The stool consistency score (a), efflux time of glass beads (b), and the volume threshold (c) are used to evaluate the intestinal transit and visceral sensitivity, respectively. The rats are divided into the control group, the model group, and the abdominal massage (AM) group. ^#^*P* < 0.05, ^##^*P* < 0.01 compared with the control group and ^*∗*^*P* < 0.05, ^*∗∗*^*P* < 0.01 (*n* = 6) compared with the model group. Effects of abdominal massage on colonic histological assessment (d). The rats are divided into the control group (A), the model group (B), and the abdominal massage (AM) group (C) (×200). Effects of abdominal massage on mast cell counts of IBS-D rats (e). The rats are divided into the control group (A), the model group (B), and the abdominal massage (AM) group (C).

The stool consistency score and efflux time of glass beads were used to evaluate the intestinal transit of rats. As shown in Figures 1(a) and 1(b), the stool consistency score increased and the efflux time of glass beads decreased significantly compared with those of the control group after 2 weeks of continuous acetic acid stimulation, which indicated that the colonic transport frequency increased significantly after continuous acetic acid stimulation. Continuous abdominal massage decreased the stool consistency score and increased the efflux time of the glass beads compared with the model groups (*P* < 0.01). Next, visceral sensitivity was investigated by the volume threshold of the abdominal uplift and back arch. Compared with the control group, the volume threshold decreased significantly, which indicated increased visceral sensitivity after continuous acetic acid stimulation. After continuous abdominal massage, the volume threshold increased significantly compared with the model group (Figure 1(c)), suggesting improved visceral sensitivity with the treatment of continuous abdominal massage.

### 3.2. Effect of Abdominal Massage on Colonic Histological Assessment and Mast Cell Counts

The H&E staining results are shown in Figure 1(d). The normal rats showed intact colonic mucosa and regular gland, without inflammatory cell infiltration or interstitial edema ((A) in Figure 1(d)). After continuous acetic acid stimulation, the gland was incompact, and there was some inflammatory cell infiltration ((B) in Figure 1(d)). This condition was improved by continuous abdominal massage ((C) in Figure 1(d)). The colon tissues were stained with toluidine blue, and the model group showed more mast cells (white arrows, (B) in Figure 1(e))than the control group. The number of mast cells decreased significantly compared with that of the model group ((C) in Figure 1(e)).

### 3.3. Effect of Abdominal Massage on the Expression of CHAT, nNOS, and PGP 9.5

**Figure 2:**
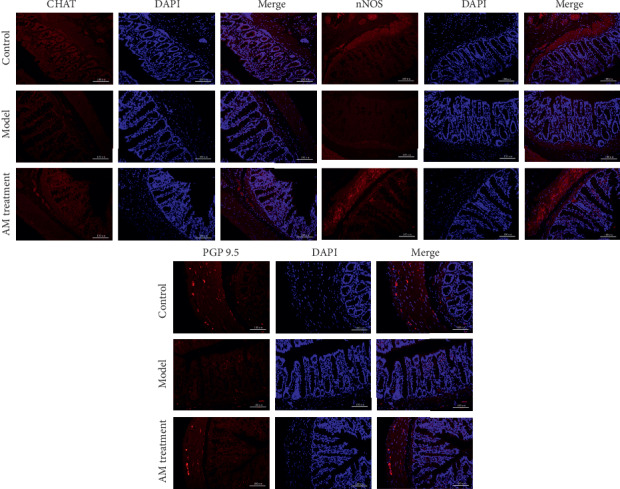
Effects of abdominal massage on the expression of CHAT, nNOS, and PGP 9.5 (immunofluorescence staining). Orange is positive protein, and DAPI (blue) is used to stain the cell nucleus (×400). The rats are divided into the control group, the model group, and the abdominal massage (AM) group.

We first investigated the protein expression of CHAT, nNOS, and PGP 9.5 by immunofluorescent staining. As shown in Figure 2, the cell nucleus was stained with DAPI (blue), and the target protein was incubated with the corresponding antibody. Compared with the control group, the positive fluorescence intensity (red) of CHAT, nNOS, and PGP 9.5 was decreased, and continuous abdominal massage increased the positive fluorescence intensity (orange) compared with the model group, which indicated higher expression of CHAT, nNOS, and PGP 9.5 by continuous abdominal massage.

**Figure 3:**
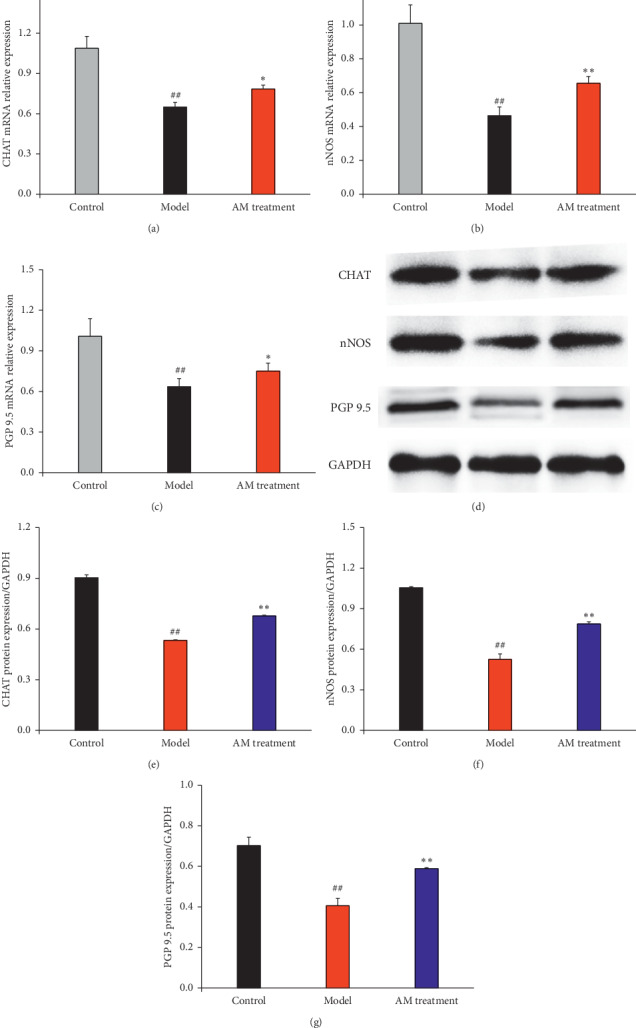
Effects of abdominal massage on the mRNA and protein expressions of CHAT, nNOS, and PGP 9.5. The mRNA and protein expressions of CHAT (a), nNOS (b), and PGP 9.5 (c). The rats are divided into the control group, the model group, and the abdominal massage (AM) group. ^#^*P* < 0.05, ^##^*P* < 0.01 compared with the control group and ^*∗*^*P* < 0.05, ^*∗∗*^*P* < 0.01 (*n* = 4) compared with the model group. The protein expression is observed by western blot (d). The rats are divided into the control group, the model group, and the abdominal massage (AM) group. The western blot film is scanned, and the intensity (CHAT (e), nNOS (f), and PGP 9.5 (g)) is quantified by Image J version 1.51n and normalized to the corresponding GAPDH intensity and the controls. ^#^*P* < 0.05, ^##^*P* < 0.01 compared with the control group and ^*∗*^*P* < 0.05, ^*∗∗*^*P* < 0.01 compared with the model group.

The mRNA expressions of CHAT, nNOS, and PGP 9.5 were also investigated by real-time RT-PCR (Figures 3(a)–3(c)). Compared with the control group, the mRNA expressions of CHAT, nNOS, and PGP 9.5 were significantly downregulated, and continuous abdominal massage could significantly upregulate the mRNA expression of CHAT, nNOS, and PGP 9.5, compared with the model group (*P* < 0.05 or *P* < 0.01). Next, the protein expressions of CHAT, nNOS, and PGP 9.5 were verified using western blot. Compared with the control group, the expressions of CHAT, nNOS, and PGP 9.5 were significantly downregulated (Figures 3(e)–3(g), *P* < 0.01). The expressions of CHAT, nNOS, and PGP 9.5 were upregulated significantly after the treatment of continuous abdominal massage compared with the model group (*P* < 0.01).

### 3.4. Effects of Abdominal Massage on the Ultrastructure of Enteric Glial Cells

**Figure 4:**
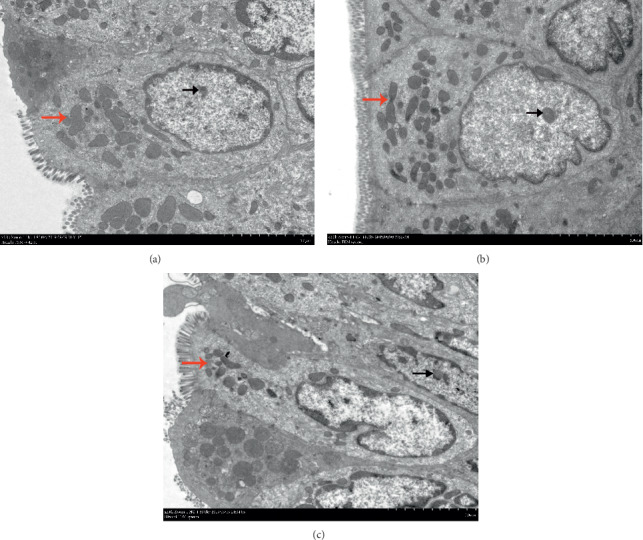
Effect of abdominal massage on ultrastructure alterations of mucosal EGCs in the colon of IBS-D rats. The rats are divided into the control group (a), the model group (b), and the abdominal massage (AM) group (c). The heterochromatin is marked with black arrows, and the mitochondria are marked with red arrows.

To investigate whether the enteric glial cells were abnormal in IBS-D, the ultrastructure of enteric glial cells was observed by transmission electron microscopy (TEM) (Figure 4). Compared with the control group, the ultrastructure of enteric glial cells in IBS-D rats showed remarkable variation with more mitochondria (red arrows) and less heterochromatin (black arrows) (Figures 4(a) and 4(b)). Continuous abdominal massage could decrease the number of mitochondria and increase the level of heterochromatin significantly compared with the model group. The results showed that there was abnormal activity of enteric glial cells in IBS-D rats, and continuous abdominal massage could improve the condition.

### 3.5. Effects of Abdominal Massage on the PI3K-Akt Signaling Pathway

**Figure 5:**
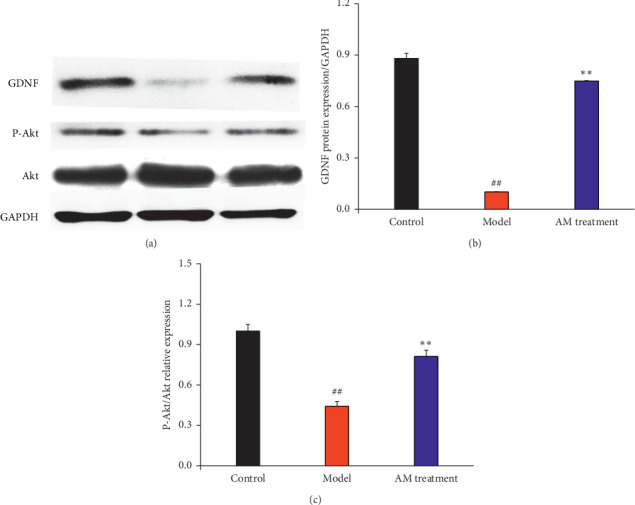
Effect of abdominal massage on the PI3K-Akt signal pathway. Western blotting analysis of GDNF, P-Akt, and Akt expression (a). The intensity is quantified by Image J version 1.51n and normalized to the corresponding GAPDH intensity and the controls. ^#^*P* < 0.05, ^##^*P* < 0.01 compared with the control group and ^*∗*^*P* < 0.05, ^*∗∗*^*P* < 0.01 compared with the model group (b and c).

Glial cell line-derived neurotrophic factor (GDNF) is important for activating the phosphoinositide 3-kinase (PI3K) pathway; therefore, we first investigated the protein expression of GDNF by WB (Figure 5). Compared with the control group, the expression of GDNF was downregulated in IBS-D rats, and continuous abdominal massage significantly upregulated the expression of GDNF (Figures 5(a) and 5(b), *P* < 0.01). The expression of P-Akt was significantly downregulated, and the expression of total Akt was upregulated slightly in IBS-D rats, and continuous abdominal massage could significantly upregulate the expression ratio of P-Akt/Akt (Figure 5(c), *P* < 0.01).

## 4. Discussion

Although drugs were used to treat IBS, there were also concomitantly more side effects. As a complementary therapy, abdominal massage has received more attention in the clinical setting. Abdominal massage has already been used for treating functional gastrointestinal disorders, including functional dyspepsia [18] and chronic constipation [19]. However, there have been few reports about the effect of abdominal massage on IBS.

The disorder of intestinal transit was one major evaluation index for IBS-D, and the IBS-D rats showed dysfunction of intestinal transit with higher stool consistency score and shorter efflux time of glass beads than control rats. Moreover, the IBS-D rats showed lower visceral hypersensitivity compared with the control rats, which was in accordance with the clinical observations in IBS-D patients [20]. Continuous abdominal massage could not only decrease the stool consistency score and increase the efflux time of glass beads but also enhance visceral hypersensitivity. These results suggest that continuous abdominal massage could improve the intestinal transit and visceral hypersensitivity of IBS-D rats.

Although the detailed pathological mechanism of IBS remains unclear, both stress and psychological factors are regarded as the main factors affecting IBS [21]. It was reported that there were increased mast cells observed in the colon and rectum mucosa of IBS patients, and the degree of increasing mast cells was positively correlated with the severity of IBS symptoms [22]. Mast cells play an important role in the pathophysiological process of IBS, which are probably an intermediate medium between the intestinal tract and the nervous system [23]. Continuous abdominal massage could significantly decrease mast cell counts, which alleviates the severity of IBS symptoms.

As the ENS plays an important role in GI motility, we inferred that enteric neurons in the ENS are also probably affected by continuous abdominal massage. Indeed, our current study showed that continuous abdominal massage restored the lost enteric neurons with both inhibitory nNOS neurons and excitatory CHAT neurons in the colon. Our results showed that continuous abdominal massage could not only upregulate the mRNA and protein expressions of nNOS and CHAT but also upregulate the mRNA and protein expression of protein gene product 9.5 (PGP9.5). These results suggest that continuous abdominal massage might balance different subtypes of enteric neurons to repair the impaired ENS and ameliorate GI motility.

Enteric glial cells (EGCs), with a function similar to astrocytes in the brain, can form an extensive cell network throughout the intestinal wall [24]. The EGC network, which connects the intestinal epithelium and the submucosal ENS, was reported to play an important role in the maintenance of intestinal homeostasis [25, 26]. A previous study revealed that colon-derived EGCs in IBS patients had a decrease in the integrated optical density of heterochromatin and an increased number of mitochondria [27], which suggested more transcriptions [28] and elevated protein synthesis [29], respectively. In this study, the ultrastructural alterations of EGCs were investigated by TEM, and the results showed that continuous abdominal massage could increase the density of heterochromatin and decrease the number of mitochondria. Recent studies have shown that the function of EGCs is affected by many factors, such as proinflammatory cytokines [30], bacteria [31], and neurotransmitters [32]. EGCs can maintain dynamic equilibrium by modulating intestinal barrier function, mucosal immunity, and enteric neurotransmission by releasing various substances [26]. GDNF has been identified to be expressed in EGCs [33].

Glial cell line-derived neurotrophic factor (GDNF) is an important member of the neurotrophic factor family, and high concentrations of GDNF were detected in the colon of the adult rat. There is evidence suggesting that GDNF could contribute to intestinal neuron differentiation, migration, proliferation, and survival [34–36]. PI3K/Akt, the downstream signaling pathway of GDNF, is an important pathway for the survival of intestinal neurons. Few studies have reported on the effect of abdominal massage on GDNF and its downstream signal pathways. In the present study, it is the first time to investigate the effect of abdominal massage on the expression of GDNF and the change of the PI3K/Akt pathway in the ENS. The results showed that, after continuous abdominal massage on IBS-D rats, abdominal massage could induce regeneration of lost enteric neurons by GDNF and PI3k/Akt signal pathways in IBS-D rats.

In the present study, continuous abdominal massage could improve the intestinal transit and visceral hypersensitivity of IBS-D rats and decrease the mast cell counts to alleviate the severity of IBS symptoms. Continuous abdominal massage could affect enteric neurons in the ENS by upregulating the expression of nNOS, CHAT, and PGP9.5. Further research indicated that continuous abdominal massage induced regeneration of lost enteric neurons by GDNF and PI3k/Akt signaling pathways in IBS-D rats. These results suggest that continuous abdominal massage is a suitable complementary therapy for treating IBS.

## 5. Conclusions

In the present study, continuous abdominal massage could improve the intestinal transit and visceral hypersensitivity of IBS-D rats and decrease the mast cell counts to alleviate the severity of IBS symptoms. The continuous abdominal massage could affect the enteric neurons in the ENS by upregulating the expressions of nNOS, CHAT, and PGP9.5. Further research indicated that continuous abdominal massage induced regeneration of lost enteric neurons by GDNF and PI3k/Akt signal pathways in IBS-D rats. These results suggested that continuous abdominal massage is the suitable complementary therapy for treating IBS.

## Figures and Tables

**Table 1:** AWR score standard.

Score	Description
0	No behavioral response to CRD
1	Simple head movement followed by immobility
2	Contraction of abdominal muscles
3	Lifting of the abdomen
4	Arching of the body and lifting of pelvic structures

## Data Availability

The datasets analyzed during the current study are available from the corresponding author upon reasonable request.

## Supplementary Materials

Supplementary Materials Table S1: the Bristol Stool Form. Table S2: the real-time RT-PCR oligonucleotide primers.
