# Supplementary material for: Abdominal Massage Reduces Visceral Hypersensitivity via Regulating GDNF and PI3K/AKT Signal Pathway in a Rat Model of Irritable Bowel Syndrome
Source: Evid Based Complement Alternat Med. 2020 Jun 5;2020:3912931. doi: 10.1155/2020/3912931 (PMC7293735; doi:10.1155/2020/3912931)
Supplement: Supplementary Materials — Table S1: the Bristol Stool Form. Table S2: the real-time RT-PCR oligonucleotide primers. [file 3912931.f1.docx]

Table S1. The Bristol Stool Form

| Type | Description |
| --- | --- |
| 1 | Separate hard lumps like nuts (difficult to pass) |
| 2 | Sausage shaped but lumpy |
| 3 | Like a sausage but with cracks on its surface |
| 4 | Like a sausage or snake, smooth and soft |
| 5 | Soft blobs with clear-cut edges (passed easily) |
| 6 | Fluffy pieces with ragged edges, a mushy stool |
| 7 | Watery, no solid pieces, entirely liquid |

Table S2. The real-time RT-PCR oligonucleotide primers.

| Gene | Primer | Sequence (5’-3’) | PCR product (bp) | |
| --- | --- | --- | --- | --- |
| GAPDH  (NM_ 017008) | Forward  Reverse | GTATGACTCTACCCACGGCAAGT  TTCCCGTTGATGACCAGCTT | 238 | |
| nNOS  (NM_ 052799) | Forward  Reverse | CGATCGGCCCTTGGTAGA  AGGCAATGCCCCTGAGAAC | 305 | |
| CHAT  (XM_ 224626) | Forward  Reverse | CAGAAGGCTGAGGTGGAAATG  CTGCTGAGGAGGCGAGATG | 1425 | |
| PGP9.5  (NM_ 017237) | Forward  Reverse | CCCTGAAGACAGAGCCAAGTG  GAGTCATGGGCTGCCTGAA | 428 | |
|  | | | |  |
